# Untargeted metabolomic profiling of sepsis-induced cardiac dysfunction

**DOI:** 10.3389/fendo.2023.1060470

**Published:** 2023-02-16

**Authors:** Yan Cao, Zhengyu Liu, Wenfeng Ma, Chen Fang, Yanfang Pei, Yingxia Jing, Jie Huang, Xiaotong Han, Weiwei Xiao

**Affiliations:** ^1^ Department of Emergency, Hunan Provincial People’s Hospital, The First Affiliated Hospital of Hunan Normal University, Changsha, Hunan, China; ^2^ Clinical Research Center for Emergency and Critical Care in Hunan Province, Hunan Provincial People’s Hospital, The First Affiliated Hospital of Hunan Normal University, Changsha, China; ^3^ Department of Cardiology, Hunan Provincial People’s Hospital, The First Affiliated Hospital of Hunan Normal University, Changsha, China; ^4^ Clinical Research Center for Heart Failure of Hunan Province, Hunan Provincial People’s Hospital, The First Affiliated Hospital of Hunan Normal University, Changsha, China; ^5^ Emergency Department, The First Affiliated Hospital of Hunan Normal University, Changsha, China; ^6^ Institute of Emergency Medicine, Hunan Provincial People’s Hospital, The First Affiliated Hospital of Hunan Normal University, Changsha, China

**Keywords:** sepsis, cardiac dysfunction, metabolomics, diagnosis, prognosis

## Abstract

**Objective:**

Sepsis is a life-threatening condition secondary to infection that evolves into a dysregulated host response and is associated with acute organ dysfunction. Sepsis-induced cardiac dysfunction is one of the most complex organ failures to characterize. This study performed comprehensive metabolomic profiling that distinguished between septic patients with and without cardiac dysfunction.

**Method:**

Plasma samples collected from 80 septic patients were analysed by untargeted liquid chromatography-mass spectrometry (LC-MS) metabolomics. Principal component analysis (PCA), partial least squares discrimination analysis (PLS-DA), and orthogonal partial least square discriminant analysis (OPLS-DA) were applied to analyse the metabolic model between septic patients with and without cardiac dysfunction. The screening criteria for potential candidate metabolites were as follows: variable importance in the projection (VIP) >1, *P* < 0.05, and fold change (FC) > 1.5 or < 0.7. Pathway enrichment analysis further revealed associated metabolic pathways. In addition, we constructed a subgroup metabolic analysis between the survivors and non-survivors according to 28-day mortality in the cardiac dysfunction group.

**Results:**

Two metabolite markers, kynurenic acid and gluconolactone, could distinguish the cardiac dysfunction group from the normal cardiac function group. Two metabolites, kynurenic acid and galactitol, could distinguish survivors and non-survivors in the subgroup analysis. Kynurenic acid is a common differential metabolite that could be used as a candidate for both diagnosis and prognosis for septic patients with cardiac dysfunction. The main associated pathways were amino acid metabolism, glucose metabolism and bile acid metabolism.

**Conclusion:**

Metabolomic technology could be a promising approach for identifying diagnostic and prognostic biomarkers of sepsis-induced cardiac dysfunction.

## Introduction

In critically ill patients, sepsis remains a common condition that is associated with high mortality and substantial morbidity (1). Cardiac dysfunction caused by sepsis, referred to as sepsis-induced cardiomyopathy, has long been a subject of interest because mortality can be greatly increased if sepsis is combined with this complication (2). Thus, it is critical to assess the probability of septic cardiomyopathy and predict clinical outcome in the early stage of patient admission. However, the prognostic and therapeutic importance of physiological changes observed in sepsis-induced cardiac dysfunction remains poorly understood. In addition, although early application of ultrasonography is helpful for the diagnosis of septic cardiomyopathy, it lacks the basis for prognostic prediction. There is also no robust evidence to identify the association of troponin and brain natriuretic peptide (BNP) with septic cardiomyopathy (3). Troponins, including troponin T and troponin I, are widely used markers for myocardial injury. The proportion of patients with elevated troponin T is up to 80% in sepsis (4), but no evidence has been found to identify the relationship between left ventricular (LV) dysfunction and troponin T (5). Another study reported that troponin I level was also not an independent predictor of mortality rate in sepsis (6). BNP, secreted by cardiomyocytes of the ventricle, is dependent on LV filling pressures and myocardial wall stretch (7). Julien et al. reported that BNP plasma levels are possibly useful for detecting myocardial dysfunction, but persistence of high BNP levels is associated with age and acute kidney injury (8). These confounders should also be considered.

Metabolomics technology is an emerging omics science developed after genomics and proteomics. As an important part of systems biology, this technology qualitatively and quantitatively analyses a broad spectrum of small molecule metabolites, especially energy metabolites, in organisms (9). The metabolome influences cellular physiology by regulating other levels of “omics”, including the genome, epigenome, transcriptome and proteome (10). Recent advances in metabolomic technology have led to its increasing application in biomedicine. In particular, the application of metabolomics provides a strategic advantage for elucidating the mechanism of disease, discovering biomarkers, and innovating new therapeutics (11).

Emerging studies highlight the potential application of metabolomics technology in sepsis (12). Feng and colleagues reported that sepsis-induced acute kidney injury is accompanied by an increased oxygen consumption, systemic aerobic and anaerobic metabolism, and abnormal fatty acid metabolism (13). A preliminary study demonstrated that 2-ethyl-2-hydroxybutyric acid regulates the expression of programmed cell death protein-1 on the surface of CD4+ T cells through the action of interleukin-2 or lactate, thereby affecting the prognosis of septic patients (14). However, to our knowledge, no study has been reported to clarify metabolomics changes in sepsis-induced cardiac dysfunction.

In this study, nontargeted metabolomic profiling was utilized to investigate metabolomic alterations in patients with sepsis-induced cardiac dysfunction and to provide evidence for early metabolic biomarkers for diagnosis and prognosis. Moreover, related metabolic pathways were also explored.

## Materials and methods

### Study design and participants

Between November 2020 and March 2022, adults who had been admitted to the emergency department of Hunan Provincial People’s Hospital were selected for the study. Patients with sepsis were included in the study within the first 24 hours after admission. The inclusion criteria of patients were based on Sepsis 3.0 in the Third International Consensus Conference. Sepsis was defined as life-threatening organ dysfunction induced by a dysregulated host response to infection and a Sequential Organ Failure Assessment (SOFA) score ≥ 2 (15). Sepsis-induced cardiac dysfunction was defined as impaired but reversible cardiac dysfunction under echocardiography, including LV systolic dysfunction, LV diastolic dysfunction, and right ventricle (RV) systolic dysfunction (16). Patients who met the exclusion criteria were as follows: (1) age < 18 years old; (2) pregnancy; and (3) history of heart disease, such as acute coronary ischaemia, LV insufficiency, dilated cardiomyopathy, hypertrophic cardiomyopathy, valvular heart disease, or recurrent arrhythmia (5). The study complied with the guidelines of the Declaration of Helsinki and the Conference for Coordination of Clinical Practice and was approved by the Ethics Committee of Hunan Provincial People’s Hospital. Informed consent was obtained from all participants.

### Blood sampling

Fasting venous blood was obtained from patients diagnosed with sepsis within 24 hours of admission. Blood samples were collected in ethylenediaminetetraacetic acid (EDTA) anticoagulant tubes and centrifuged for 10 min at 4°C and 3,000 rpm to obtain plasma. The fasting plasma samples were stored in a -80°C freezer and kept frozen until metabolomic analysis.

### Metabolite extraction and data processing

One hundred microlitres of the plasma sample was transferred to a 1.5 mL Eppendorf tube, and 20 μL of L-2-chlorophenylalanine (0.3 mg/mL) was dissolved in methanol as an internal standard. The tube was vortexed for 10 seconds. Subsequently, 300 μL of protein precipitant (methanol and acetonitrile, 2:1, vol/vol) was added, and the mixture was vortexed for 1 min. Then, the whole sample was sonicated for 10 min in an ice-water bath and stored at -20°C for 30 min. The extract was centrifuged at 4°C (13,000 rpm) for 10 min. The supernatant of each sample was collected. Quality control samples (QCs) were prepared by mixing equal volumes of extracts from all samples.

An ACQUITY UPLC I-Class system (Waters Corporation, Milford, USA) and a VION IMS QTOF mass spectrometer (Waters Corporation, Milford, USA) were used to analyse the metabolic profiles in both ESI positive and ESI negative ion modes. The target compounds were separated by an ACQUITY UPLC BEH C18 column (1.7 μm, 2.1 × 100 mm) at 45°C with 2 μL sample injection. Water and acetonitrile, both containing 0.1% formic acid, were used as mobile phases A and B, respectively. The gradient was set as follows: 0–2 min, 5% B; 4 min, 30% B; 8 min, 50% B; 10 min, 80% B; 14 min, 100% B; 15 min, 100% B; 15.1 min, 5% B and 16 min, 5% B. The flow rate was 0.35 mL/min, and the column temperature was 45 °C.

Primary and secondary mass spectrometry data were collected by a VION IMS QTOF mass spectrometer. The parameters of mass spectrometry were set as follows: a low-energy scan (CE 4 eV) and a high-energy scan (CE ramp 20–45 eV) to fragment the ions. Argon was used as the collision-induced dissociation gas; scan time: 0.2 s; interscan delay: 0.02 s; capillary voltage, 1 kV (negative mode) or 2 kV (positive mode); capillary voltage: 2.5 kV; cone voltage: 40 V; source temperature: 115°C; desolvation gas temperature: 450°C; and desolvation gas flow, 900 L/h.

The original data were analysed by Progenesis QI V2.3 (Nonlinear Dynamics, Newcastle, UK) software for baseline filtering, peak recognition, integration, retention time correction, peak alignment and normalization. Compound identification was based on the precise mass-to-charge ratio (m/z), MS2 fragments, and isotopic distribution using the Human Metabolome Database (HMDB) or Metlin, and self-built databases to do qualitative analysis. The metabolites in the self-built database are all established by standards, which contain retention time, first-level accurate mass information and second-level mass spectrum fragment information. The module pathway analysis was based on the KEGG database.

### Statistical analysis

Clinical data were analysed using SPSS Statistics 25 software (IBM^®^, Armonk, NY, USA). Data are represented as the mean ± standard deviation or median and interquartile range. Continuous variables were compared using Student’s t test or the Mann−Whitney U test. Student’s t test is used when two samples are small and meet the conditions of normal distribution and homogeneity of variance. The Mann−Whitney U test was used when the samples did not meet the conditions of normal distribution and homogeneity of variance. Categorical variables between the two groups were compared by Fisher’s exact probability method. *P* < 0.05 was considered statistically significant.

The metabolic profiles were imported into R for principal component analysis (PCA) to observe the overall distribution among the samples and the stability of the entire analysis process. Partial least squares discriminant analysis (PLS-DA) and orthogonal partial least squares discriminant analysis (OPLS-DA) were used to distinguish differential metabolites between groups. To prevent overfitting, 7-fold cross-validation and 200 response permutation tests were utilized to evaluate the quality of the model. Variable importance of projection (VIP) values obtained from the OPLS-DA model were used to rank the overall contribution of each variable to group discrimination. A two-tailed Student’s t test was further used to verify whether the differences in metabolites between groups were significant. Differential metabolites were selected with VIP >1.0, *P* < 0.05, and fold change (FC) >1.5 or <0.7. Binary logistic regression analysis was constructed to screen independent risk factors. Receiver operating characteristic (ROC) curves were constructed to evaluate the diagnostic ability of differential metabolites between the tested groups.

## Results

### Study design and clinical synopsis

**Figure 1:**
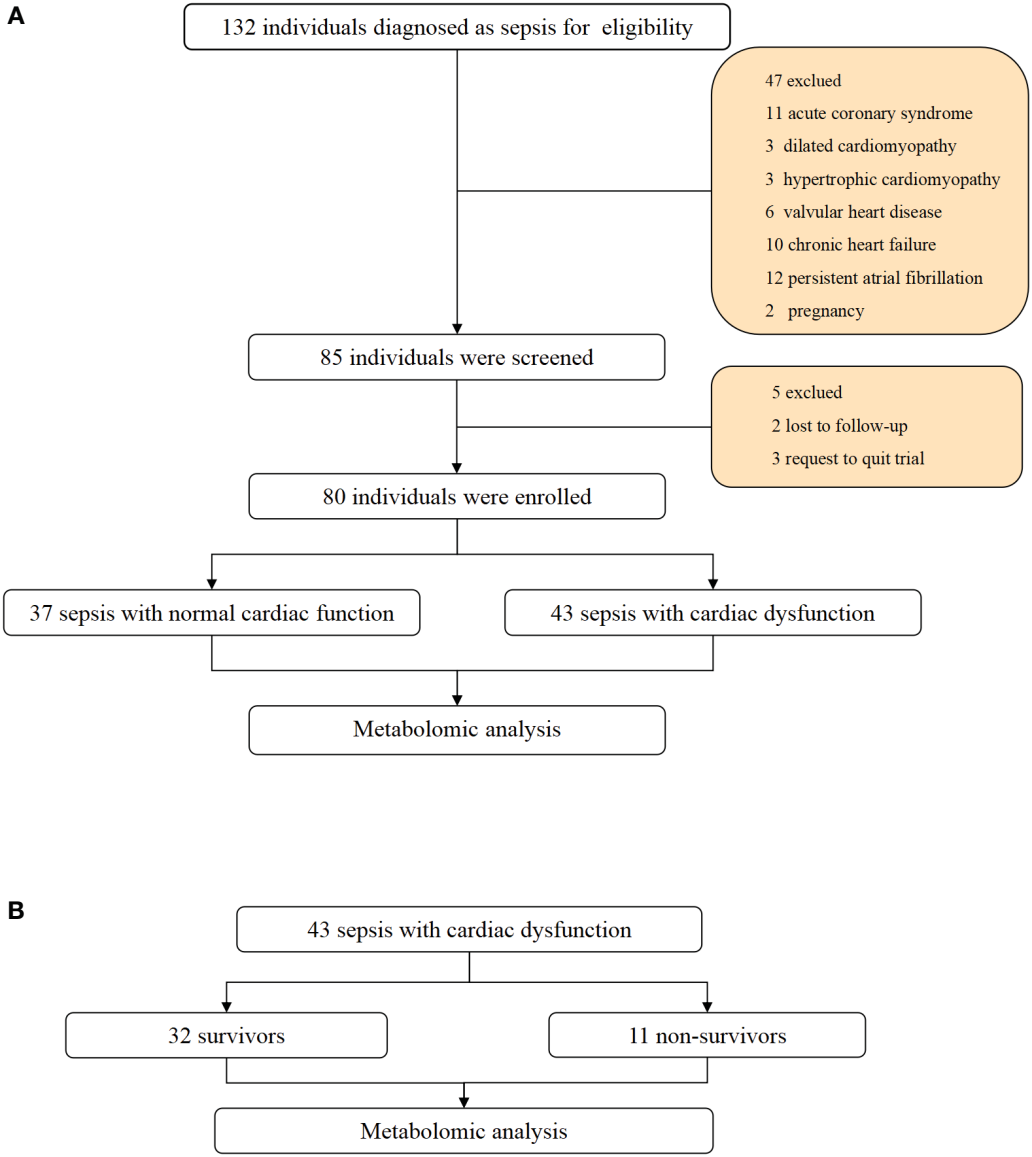
Flowchart and study design. **(A)** Flowchart of individuals enrollment and study design in total patients with sepsis. **(B)** flowchart of subgroup analysis between survivors and non-survivors in sepsis with cardiac dysfunction.

Initially, a total of 132 patients were enrolled. Based on the exclusion criteria, 37 patients with normal cardiac function and 43 patients with cardiac dysfunction were eventually included in our study. The flow chart is shown in **Figure 1**.

The clinical characteristics are shown in **Table 1**. Cardiac dysfunction is common in sepsis, affecting 54% (n=43) of patients. No significant differences in sex, age, comorbidities, or site of infection were noted between the two groups. Lactate levels and troponin I levels were higher in the cardiac dysfunction group (*P=*0.037 and *P=*0.023, respectively). N-terminal pro-brain natriuretic peptide (NT-proBNP) was elevated in the cardiac dysfunction group, but the difference was not statistically significant (*P*=0.051). The 28-day mortality was increased in the cardiac dysfunction group, although the difference was not statistically significant (*P*=0.178). ROC curves were constructed to determine the predictive value of variables in diagnosing sepsis-induced cardiac dysfunction. The AUC for lactate level (AUC 0.636, SE 95% confidence interval 0.513 to 0.759, *P* = 0.037) was equivalent to troponin I (AUC 0.647, SE 95% confidence interval 0.519 to 0.775, *P* = 0.024) and NT-proBNP (AUC 0.627, SE, 95% confidence interval 0.505 to 0.750, *P* = 0.051). The three AUCs were less than 0.75, indicating that the three variables were not robust enough for diagnosing sepsis-induced cardiac dysfunction.

**Table 1:** Clinical characteristics of subjects enrolled in the study.

Variables	Overall (N=80)	Septic patients with normal cardiac function (N=37)	Septic patients with cardiac dysfunction (N=43)	*P* value
Characteristic				
Sex (Male%)	47 (58.8)	25 (67.6)	22 (51.2)	0.208
Age (years)	59.2 ± 13.9	59.7 ± 13.6	58.7 ± 14.4	0.731
Combidities				
Hypertension	26 (32.5)	12 (32.4)	14 (32.6)	0.990
Diabetes	19 (23.8)	8 (21.6)	11 (25.6)	0.678
Chronic respiratory disease	20 (25.0)	9 (24.3)	11 (25.6)	0.897
Chronic kidney disease	8 (10.0)	2 (5.4)	6 (14.0)	0.275
Neurologic disease	7 (8.8)	2 (5.4)	5 (11.6)	0.316
Site of infection				
Lung	44 (55.0)	22 (59.5)	22 (51.2)	0.457
Gentiourinary tract	30 (37.5)	11 (29.7)	19 (44.2)	0.248
Hepatobiliary	22 (27.5)	12 (32.4)	10 (23.3)	0.359
Gastrointestine	14 (17.5)	6 (16.2)	8 (18.6)	0.779
Blood	15 (18.8)	5 (13.5)	10 (23.3)	0.390
Neural system	2 (2.5)	1 (2.7)	1 (2.3)	0.914
Soft tissue	6 (7.5)	3 (8.1)	3 (7.0)	0.848
Lactate(mmol/L)	2.0 (1.3-3.6)	1.7 (1.3-2.4)	2.2 (1.6-4.9)	0.037
Troponin I (ng/ml)	0.07 (0.02-0.84)	0.03 (0.01-0.93)	0.10 (0.04-0.92)	0.023
NT-proBNP (pg/ml)	1405.0 (526.3-5972.5)	925.0 (479.8-2596.8)	2740.0 (621.0-6910.0)	0.051
28-day mortality	16 (20.0)	5 (13.5)	11 (25.6)	0.178

Data represented as mean ± standard deviation or median and interquartile range. Continuous variables were compared using Student’s t-test or the Mann-Whitney U test.Categorical variable were compared using the chi-square test or Fisher’s exact test as appropriate.Patients with sepsis was enrolled into the study within 24 hours from admission to emergency department.

### Metabolite analysis and model validation in the normal cardiac function group and cardiac dysfunction group

**Figure 2:**
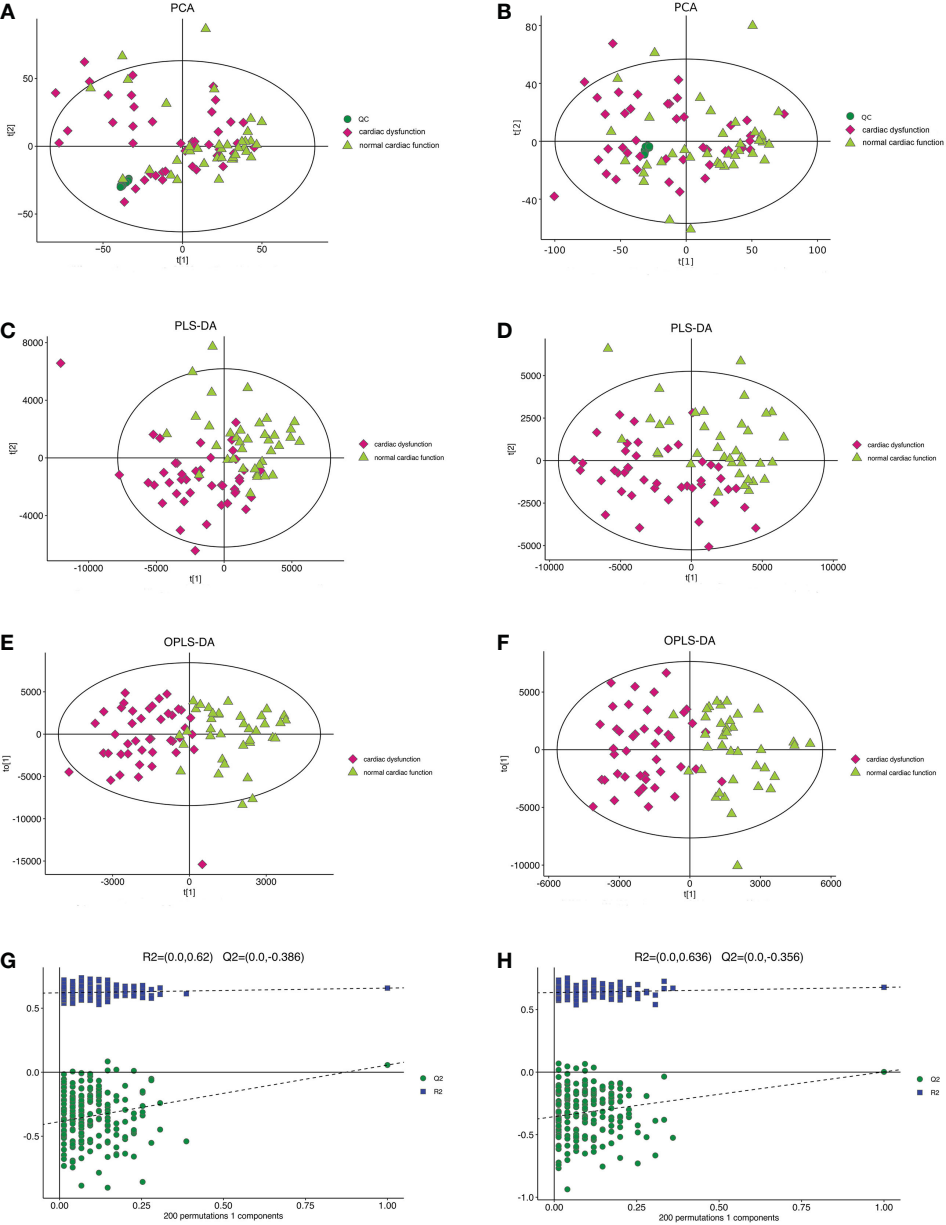
The multidimensional results in positive and negative ionization mode in total patients. **(A)** PCA score plot in positive ionization mode [R^2^X (cum) = 0.519, Q^2^ (cum) = 0.442]. **(B)** PCA score plot in negative ionization mode [R^2^X (cum) = 0.502, Q^2^ (cum) = 0.439]. **(C)** PLS-DA score plot in positive ionization mode [R^2^X (cum) = 0.219, R^2^Y (cum) = 0.756, Q^2^ (cum) = 0.563]. **(D)** PLS-DA score plot in negative ionization mode [R^2^X(cum) = 0.249, R^2^Y(cum) = 0.778 Q^2^(cum) = 0.595]. **(E)** OPLS-DA score plot in positive ionization mode [R^2^X (cum) = 0.219, R^2^Y (cum) = 0.756, Q^2^ (cum) = 0.459]. **(F)** OPLS-DA score plot in negative ionization mode [R^2^X(cum) = 0.249, R^2^Y(cum) = 0.778 Q^2^(cum) = 0.398]. **(G)** Permutation test in positive ionization mode (R^2 ^= 0.620, Q^2^= -0.386). **(H)** Permutation test in negative ionization mode (R^2 ^= 0.636, Q^2^= -0.356). PCA, principal component analysis; PLS-DA, partial least squares discrimination analysis; OPLS-DA, orthogonal partial least squares-discriminant analysis.

The PCA model in the positive and negative ion modes showed that the instrument was stable throughout the experiment. The red squares, green triangles, and green circles in the figure represent the cardiac dysfunction group, normal cardiac function group, and QC group, respectively (**Figures 2A, B**).

Then, the PLS-DA method was applied to analyse the metabolite profile: in the positive ion mode, there were significant differences between the normal cardiac function group and the cardiac dysfunction group (CV-ANOVA,F=17.796, *P*<0.001]; in the negative ion mode, the two groups also exhibited significant differences (CV-ANOVA, F=19.066, *P*<0.001) (**Figures 2C, D**). These findings indicate that the PLS-DA model could be used to distinguish septic patients with cardiac dysfunction from those with normal cardiac function.

To achieve the greatest separation of differential metabolites between the two groups, OPLS-DA was performed based on values of VIP > 1. OPLS-DA demonstrated notable separation in metabolic profiles between the cardiac dysfunction group and the normal cardiac function group (**Figures 2E, F**). The permutation chart verified the validity of the model. R^2^ and Q^2^ were generated by permutation test. When R^2^ > 0, Q^2^ < 0, it indicates that the model is reliable (17). The results of the permutation chart showed that the models were reliable (**Figures 2G, H**).

### Screening of differential metabolites between the normal cardiac function group and cardiac dysfunction group

**Figure 3:**
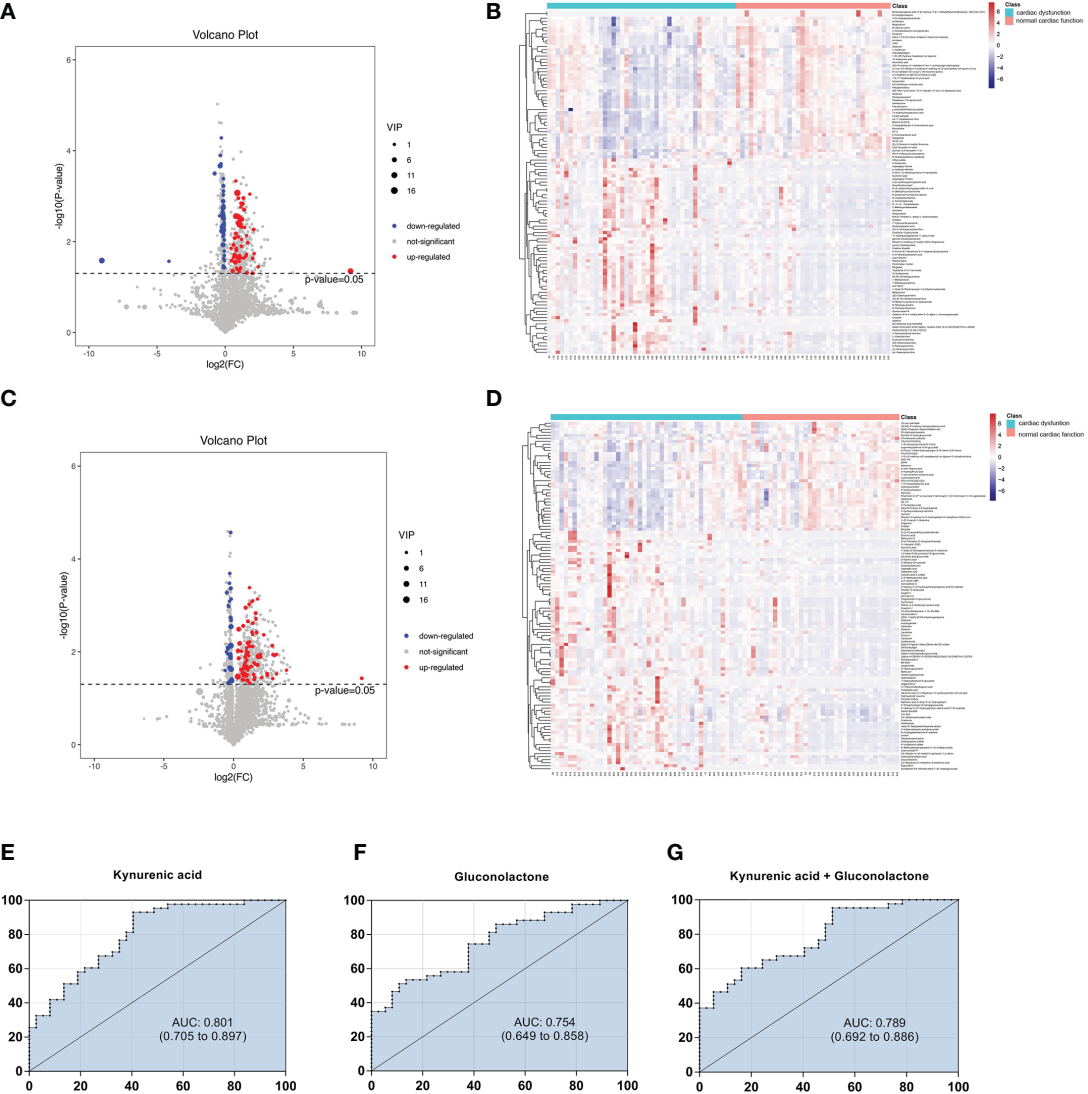
Volcano diagrams and heatmaps in total patients. **(A)** Volcano map in positive ionization mode. **(B)** Heatmap in positive ionization mode. **(C)** Volcano map in negative ionization mode. **(D)** Heatmap in negative ionization mode. In the volcano diagrams, the red dots represent up-regulated metabolites, and the gray dots represent no significant difference, and the blue dots represent down-regulated metabolites. **(E–G)** ROC curve analysis of the ability of differential metabolites to predict sepsis with cardiac dysfunction.

Univariate statistical analysis was applied to screen differential metabolites. A volcano plot was utilized to visualize the *P* value, VIP and fold change value. The red dots represent the upregulated metabolites, while the blue dots represent the downregulated metabolites (**Figures 3A, C**). The heatmap intuitively displays differential metabolites in different samples. In the heatmap of positive and negative ion modes, the Z scores of potential biomarkers were labelled in terms of the types of metabolites (**Figures 3B, D**). The most differential metabolites, which can be candidate for biomarkers, are presented in **Table 2**. The screening criteria for potential biomarkers were as follows: VIP >1, *P* < 0.05, and FC > 1.5 or < 0.7 (17). Seventeen metabolites differed significantly between the normal cardiac function group and the cardiac dysfunction group.

**Table 2:** In two ionization modes, differential metabolites between normal cardiac function group and cardiac dysfunction group.

Ionization mode Metabolites		Formula	Compound ID HMDB (or Metlin)	Normal cardiac function group *vs.* cardiac dysfunction group		
				VIP	FC	*P* value
Pos	L-Acetylcarnitine	C9H17NO4	HMDB0000201	11.359	1.901	<0.001
	Creatine	C4H9N3O2	7	5.159	1.708	0.022
	5-Aminopentanoic acid	C5H11NO2	HMDB0003355	2.740	0.591	<0.001
	N1-Methyl-4-pyridone-3-carboxamide	C7H8N2O2	HMDB0004194	1.638	1.689	0.042
	3-Hydroxy-N6,N6,N6- 4-trimethyl-L-lysine	C9H20N2O3	6234	1.227	1.731	0.013
	Quinolinic acid	C7H5NO4	HMDB0000232	1.159	2.720	0.017
	5-Methoxyindoleacetate	C11H11NO3	7016	1.049	1.625	0.010
	25-Hydroxycholesterol	C27H46O2	HMDB0006247	1.220	0.677	0.004
Neg	Gluconic acid	C6H12O7	345	9.590	3.622	0.009
	Glycochenodeoxycholic acid 3-glucuronide	C32H51NO11	HMDB0002579	4.715	0.367	0.033
	3alpha-Androstanediol glucuronide	C25H40O8	HMDB0246252	4.201	1.748	0.016
	D-Xylonic acid	C5H4N4O3	HMDB0059750	3.233	1.899	<0.001
	Pregnanediol-3- glucuronide	C27H44O8	HMDB0010318	2.821	2.068	0.008
	Uridine	C9H12N2O6	HMDB0000296	2.782	1.677	0.001
	Kynurenic acid	C10H7NO3	HMDB0000715	1.389	4.698	0.003
	5’-Phosphoribosyl-N-formylglycinamide	C8H15N2O9P	HMDB0001308	1.347	1.583	0.018
	Gluconolactone	C6H10O6	HMDB0000150	1.180	2.299	<0.001

Given VIP >1.0, P < 0.05, and FC >1.5 or < 0.7, 17 metabolites were identified.

The differential metabolites that enriched into pathways were listed in a decreasing order according to VIP.

Subsequently, we constructed binary logistic regression analysis to screen risk factors and ROC curve analysis to evaluate the predictive ability in the differential metabolites. Kynurenic acid, gluconolactone, 3-hydroxy-N6,N6,N6-trimethyl-L-lysine and 25-hydroxycholesterol were indentified as independent risk factors for sepsis-induced cardiac dysfunction, and the AUC values of kynurenic acid and gluconolactone were > 0.75 (**Supplymentary Tables 1**, **2**). The AUCs for kynurenic acid and gluconolactone were 0.801, 0.754, respectively. We also constructed ROC analysis combining kynurenic acid and gluconolactone. However, the AUC for combining kynurenic acid and gluconolactone was 0.789, which was not superior to kynurenic acid (**Figures 3E–G**).

### Differential metabolic pathways between the normal cardiac function group and cardiac dysfunction group

**Figure 4:**
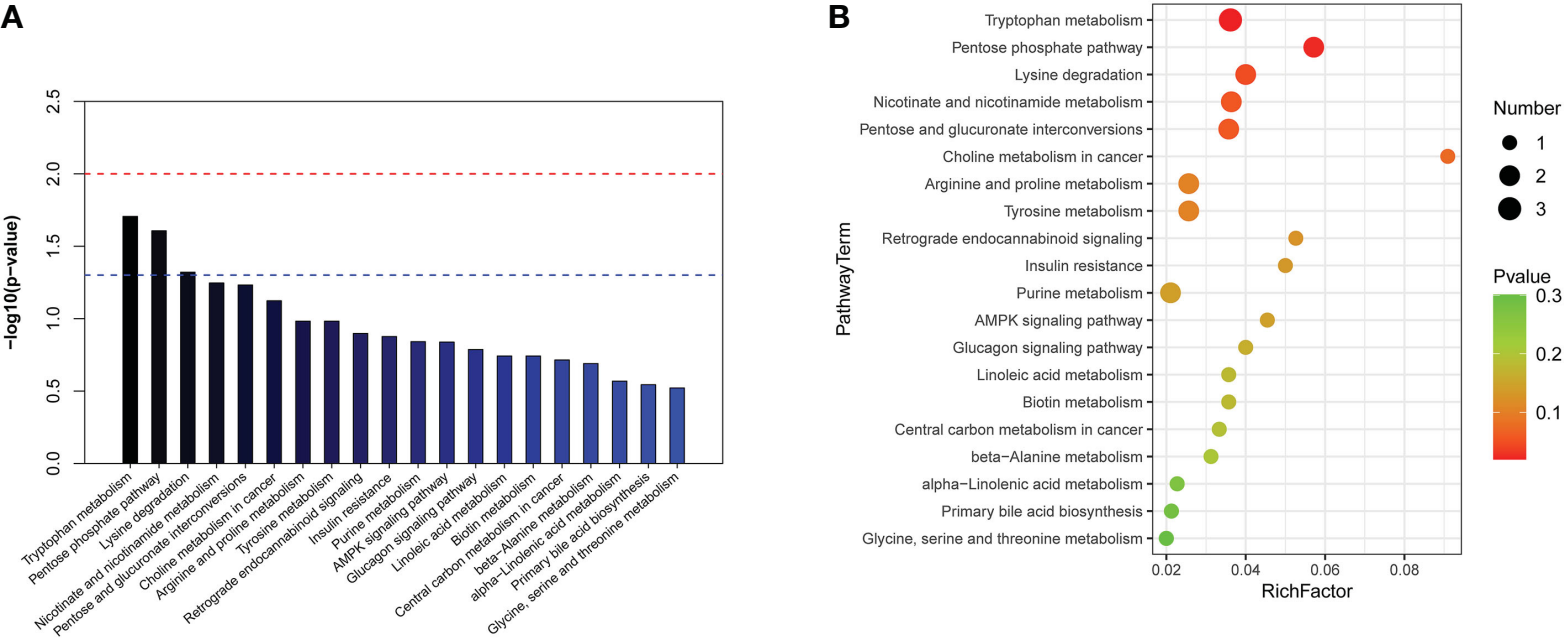
The significantly enriched pathways involved in total patients. **(A)** Top-20 metabolic pathway enrichment map.The red line indicates a P value of 0.01, and the blue line indicates a P value of 0.05. When the top of the bar is higher than the blue line, the pathway is considered to be significant. **(B)** Top-20 bubble chart. The colour changed from green to red indicates that significance increases in turn; the larger the point is, the greater the number of metabolites enriched in the pathway.

KEGG and HMDB were applied to analyse cardiac dysfunction-associated metabolites, and the results were submitted to MetaboAnalyst. In the top 20 metabolic pathway enrichment maps, significance is indicated by logarithm of the reciprocal of P value based on 10 (**Figure 4A**). In the top 20 bubble chart, the colour and size of each circle are determined by *P* values. (**Figure 4B**). The following three important metabolic pathways were identified: tryptophan metabolism, the pentose phosphate pathway, and lysine degradation. The results of pathway enrichment analysis for these differential metabolites are summarized in **Supplementary Table 3**.

### Metabolite analysis and model validation in the survivors and non-survivors of cardiac dysfunction group

**Figure 5:**
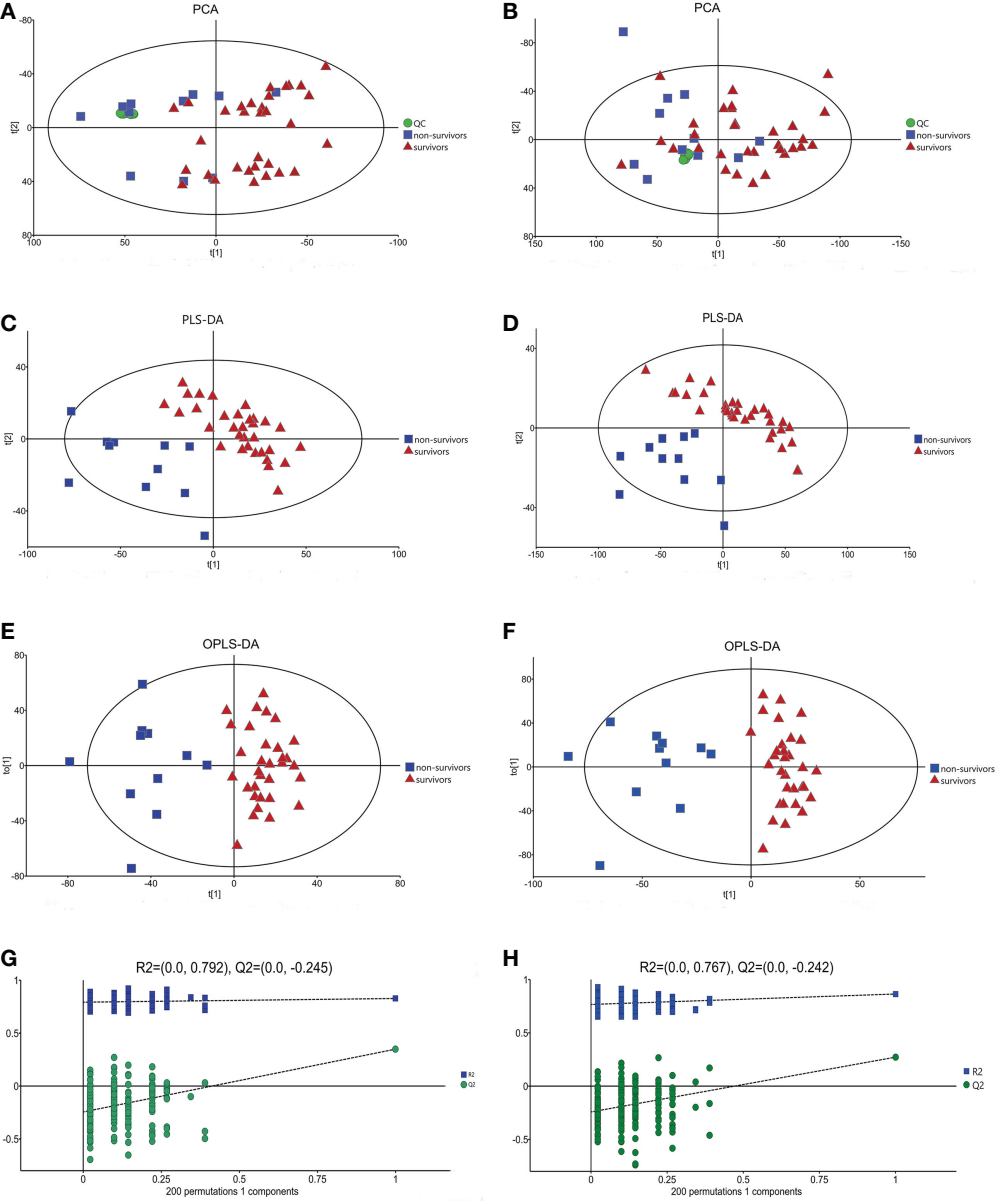
The multidimensional results in positive and negative ionization between survivors and non-surviviors of cardiac dysfunction group. **(A)** PCA score plot in positive ionization mode [R^2^X (cum) = 0.497, Q^2^ (cum) = 0.401]. **(B)** PCA score plot in negative ionization mode [R^2^X (cum) = 0.507, Q^2^ (cum) = 0.369]. **(C)** PLS-DA score plot in positive ionization mode [R^2^X (cum) = 0.409, R^2^Y (cum) = 0.787 Q^2^ (cum) = 0.582]. **(D)** PLS-DA score plot in negative ionization mode [R^2^X(cum) = 0.447, R^2^Y(cum) = 0.795, Q^2^(cum) = 0.576]. **(E)** OPLS-DA score plot in positive ionization mode [R^2^X (cum) = 0.409, R^2^Y (cum) = 0.787 Q^2^ (cum) = 0.483]. **(F)** OPLS-DA score plot in negative ionization mode [R^2^X(cum) = 0.447, R^2^Y(cum) = 0.795, Q^2^(cum) = 0.462]. **(G)** Permutation test in positive ionization mode(R^2 ^= 0.792, Q^2^= -0.245). **(H)** Permutation test in negative ionization mode(R^2 ^= 0.767, Q^2^= -0.242).

It has been reported that mortality can be increased if sepsis is combined with cardiac dysfunction (2). In our study, 28-day mortality was increased in the cardiac dysfunction group, although the difference was not statistically significant due to the small sample size. It is crucial for physicians to predict clinical outcome exactly in the early stage of patient admission; thus, clarifying the metabolic changes in non-survivors is of great importance. A comparison between the survivors and non-survivors in the cardiac dysfunction group was performed. The PCA, PLS-DA and OPLS-DA models distinguished the metabolic profiles of the survivors from those of the non-survivors. The blue squares, red triangles and green circles in the figure represent the non-survivors, survivors and QC samples, respectively. PLS-DA analysis of the metabolite profile of the plasma sample: results in the positive ion mode suggested that there were significant differences between survivors and non-survivors (CV-ANOVA, F=23.301, *P<*0.001); in the negative ion mode, the two groups also exhibited significant differences (CV-ANOVA, F=26.217, *P<*0.001). In the OPLS-DA model, permutation tests showed that the model is reliable. (**Figures 5A–H**).

### Screening of differential metabolites between the survivors and non-survivors of cardiac dysfunction group

**Figure 6:**
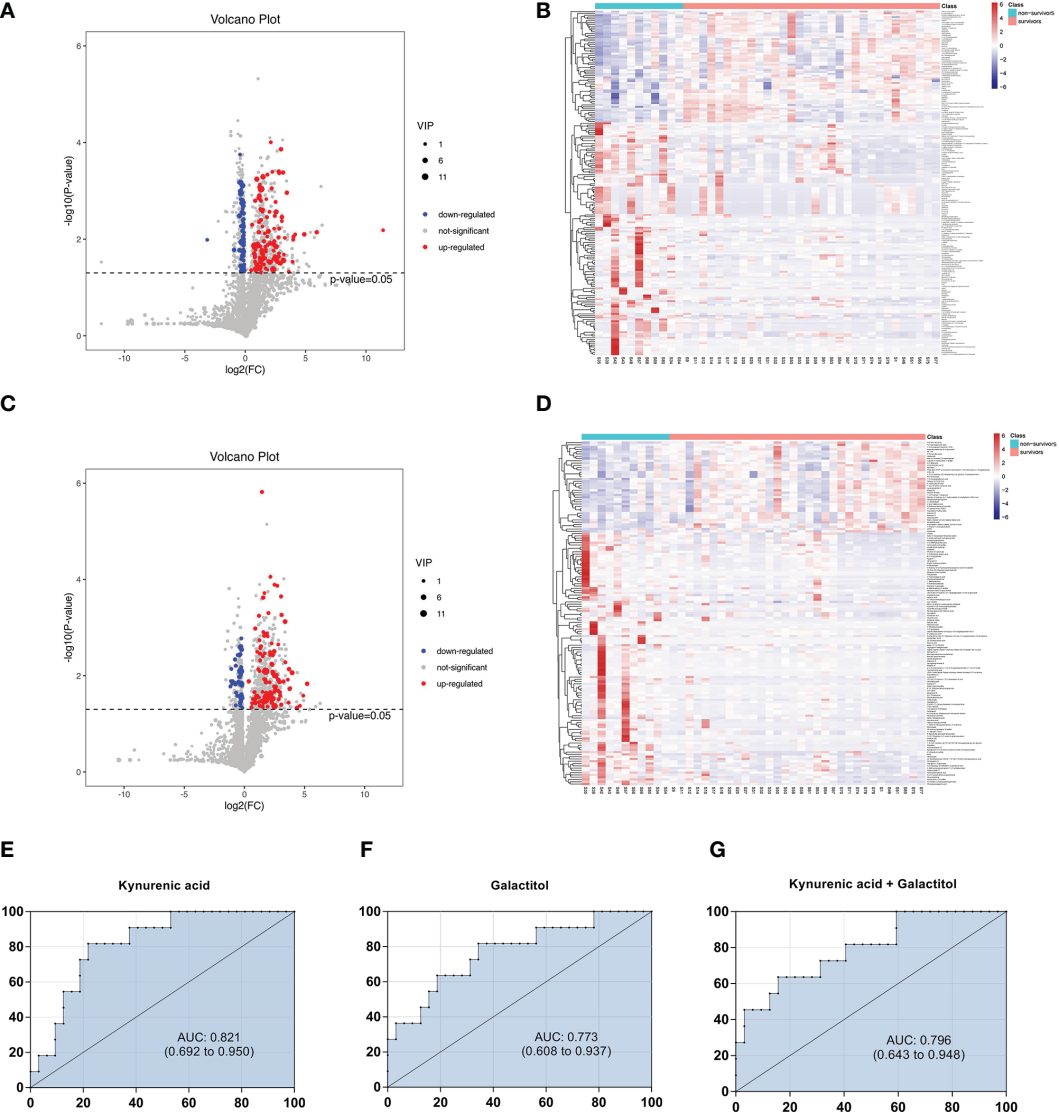
Volcano diagrams and heatmaps between survivors and non-surviviors of cardiac dysfunction group. **(A)** Volcano map in positive ionization mode. **(B)** Heatmap in positive ionization mode. **(C)** Volcano map in negative ionization mode. **(D)** Heatmap in negative ionization mode. **(E–G)** ROC curve analysis of the ability of differential metabolites to predict 28-mortality in sepsis with cardiac dysfunction.

Volcano maps and heatmaps were generated (**Figures 6A–D**). The most differential metabolites are presented in **Table 3** following these criteria: VIP >1, *P* < 0.05, and FC > 1.5 or < 0.7. Twenty-five metabolites differed significantly between the survivors and non-survivors.

**Table 3:** In two ionization modes, differential metabolites between survivors and non-survivors.

Ionization mode Metabolites		Formula	Compound ID HMDB (or Metlin)	Survivors *vs.*Non-survivors		
				VIP	FC	*P* value
Pos	L-Acetylcarnitine	C9H17NO4	HMDB0000201	9.657	1.712	0.014
	Glycocholic acid	C26H43NO6	HMDB0000138	6.943	2.566	0.026
	Taurochenodesoxycholic acid	C12H22O11	137	5.993	8.927	0.041
	Sucrose	C7H8N2O2	HMDB0004194	1.638	4.943	0.042
	Taurocholic acid	C26H45NO7S	34542	5.505	6.274	0.029
	Creatine	C4H9N3O2	7	5.002	1.935	0.034
	Phytosphingosine	C18H39NO3	7066	3.534	6.773	0.023
	Sorbitol	C6H14O6	HMDB0000247	2.740	2.742	0.002
	N-Acetylserotonin	C12H14N2O2	HMDB0001238	2.542	6.847	<0.001
	UDP-D-galacturonic acid	C15H22N2O18P2	HMDB0012302	2.449	3.118	0.025
	N1-Methyl-4-pyridone-3-carboxamide	C7H8N2O2	HMDB0004194	2.284	1.899	0.039
	N1-(alpha-D-ribosyl)-5,6-dimethyl-benzimidazole	C14H18N2O4	HMDB0011112	1.651	4.026	0.003
	S-Adenosylmethioninam-ine	C14H23N6O3S	HMDB0000988	1.650	12.208	0.048
	L-Tryptophan	C11H12N2O2	33	1.625	3.565	<0.001
	beta-D-ribosylnicotinate	C11H13NO6	HMDB0304540	1.418	5.141	0.017
	L-Histidine	C6H9N3O2	21	1.338	1.401	0.044
	S-Adenosylhomocysteine	C14H20N6O5S	HMDB0000939	1.013	3.515	0.006
Neg	Phenylacetylglutamine	C13H16N2O4	HMDB0006344	9.023	5.673	0.043
	Hippuric acid	C9H9NO3	HMDB0000714	2.469	3.321	0.035
	D-Maltose	C12H22O11	HMDB0000163	2.109	5.619	0.017
	Uridine	C9H12N2O6	HMDB0000296	1.646	1.508	0.046
	Salicylic acid	C7H6O3	HMDB0001895	1.485	2.614	0.034
	Galactitol	C6H14O6	HMDB0000107	1.310	3.640	0.001
	Kynurenic acid	C10H7NO3	HMDB0000715	1.211	2.850	0.022
	Prostaglandin I2	C20H32O5	HMDB0001335	1.206	1.764	0.036

Given VIP >1.0, P < 0.05, and FC >1.5 or < 0.7, 25 metabolites were identified.

The differential metabolites that enriched into pathways were listed in a decreasing order according to VIP.

Then, we constructed binary logistic regression analysis to screen risk factors and ROC curve analysis to evaluate the predictive ability. Only kynurenic acid and galactitol were indentified as independent risk factors for 28-day mortality in sepsis-induced cardiac dysfunction and the AUC values were > 0.75 (**Supplementary Tables 4**, **5**).The AUCs for kynurenic acid and galactitol were 0.821, 0.773, respectively. We also constructed ROC analysis combining kynurenic acid and galactitol. Whereas, the AUC for combining kynurenic acid and alactitol was 0.796, which was not superior to kynurenic acid (**Figures 6E–G**).

### Differential metabolic pathways between survivors and non-survivors in the cardiac dysfunction group

**Figure 7:**
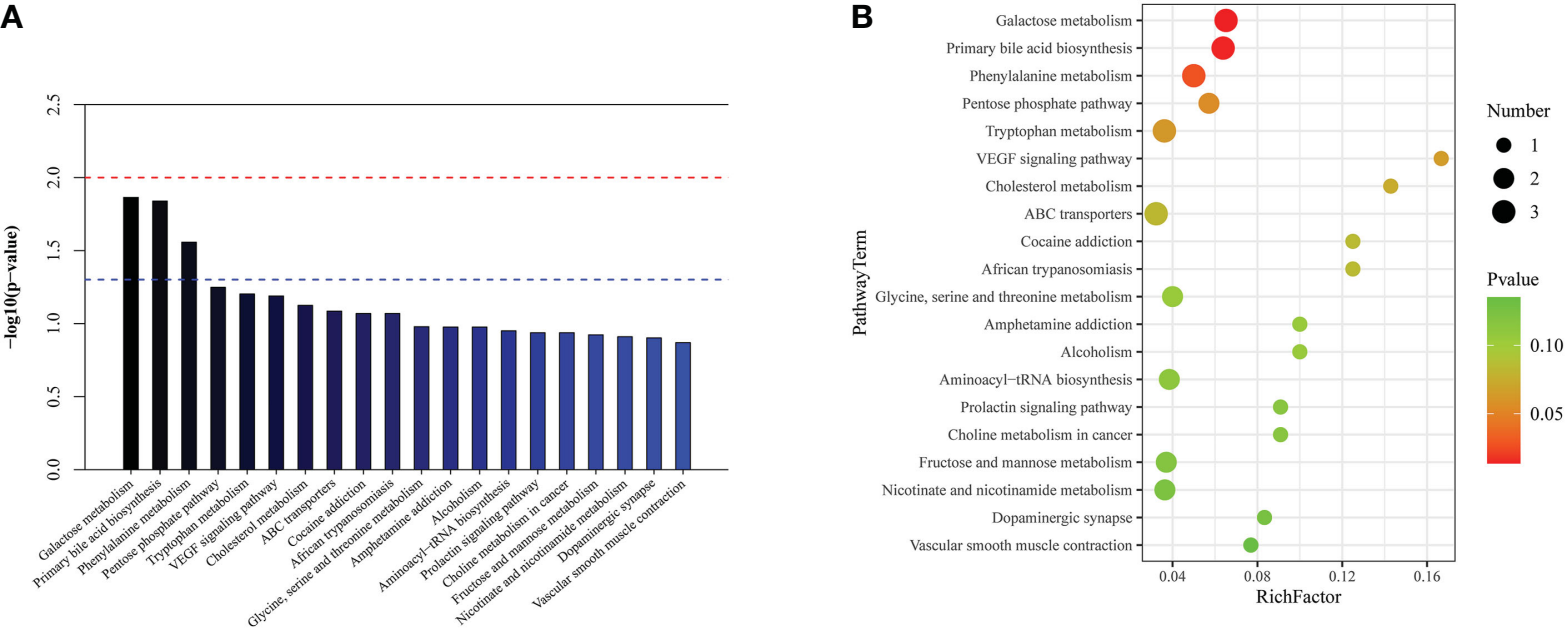
The significantly enriched pathways involved in cardiac dysfunction group. **(A)** Top-20 metabolic pathway enrichment map. **(B)** Top-20 bubble chart.

In the top 20 metabolic pathway enrichment maps and bubble chart (**Figures 7A, B**), the following three important metabolic pathways were identified as differential metabolic pathways between the survivors and non-survivors: galactose metabolism, primary bile acid biosynthesis, and phenylalanine metabolism. The results of pathway enrichment analysis for these differential metabolites are summarized in **Supplementary Table 6**.

## Discussion

Metabolomics is a rapidly developing technology that has been applied in many fields, such as biomarker investigation. To the best of our knowledge, this is the first study to perform metabolic analysis for septic patients with cardiac dysfunction. We first identified that key metabolites were changed during the development of sepsis-induced cardiac dysfunction. Two metabolite markers, kynurenic acid, and gluconolactone could distinguish the cardiac dysfunction group from the normal cardiac function group. Accordingly, we also discovered three important metabolic pathways related to sepsis-induced cardiac dysfunction, including tryptophan metabolism, pentose phosphate pathway, and lysine degradation. Next, we performed a comparison between the survivors and non-survivors in the cardiac dysfunction group. Two metabolites, including kynurenic acid and galactitol, could distinguish survivors and non-survivors in patients with sepsis-induced cardiac dysfunction. The crucial metabolic pathways are galactose metabolism, primary bile acid biosynthesis, and phenylalanine metabolism.

The most important finding was the identification of kynurenic acid as a potential metabolite biomarker for diagnosing sepsis-induced cardiac dysfunction and predicting poor outcomes. Kynurenic acid is a key degradation metabolite of tryptophan through the kynurenine pathway. Compared with kynurenine, kynurenic acid is not metabolized and stable in solution (18). Hence, kynurenic acid is reported to be a potential candidate laboratory biomarker of metabolic diseases as atherosclerosis and diabetes (19). Tryptophan is an essential amino acid that can only be provided from nutrition intake. Its metabolites play key roles in a variety of physiological processes, from cell growth and maintenance to coordinating the organism’s response to the environment (20). Tryptophan catabolism by the kynurenine pathway is a pivotal metabolic pathway closely associated with the innate immune system (21). In the early stage of sepsis, indoleamine 2,3-dioxygenase 1 (IDO1), a key enzyme that converts tryptophan to kynurenine, is activated and enhances proinflammatory effects that may result in severe tissue damage and septic shock (22). Afterwards, the accumulation of kynurenine and its metabolites, such as kynurenic acid, can induce immunotolerance or even immunoparalysis through the expansion of regulatory T cells (Tregs) and T helper 2 (Th2) cells and with the mitigation of inflammatory Th1 and Th17-mediated responses (23). Immunosuppression may contribute to the inability to overcome secondary infection and late mortality. These findings may explain why kynurenic acid levels were remarkably elevated in the cardiac dysfunction group and the non-survivors in this group. To our knowledge, this is the first study to find increased kynurenic acid in sepsis-induced cardiac injury. However, the underlying mechanism needs further investigation.

Our data also revealed that another important metabolite, gluconolactone, in the pentose phosphate pathway could be used to distinguish the cardiac dysfunction group and normal cardiac function group. Gluconolactone is considered to be a free radical scavenger. It relieves ischaemia/perfusion-induced cardiac injury although activation of extracellular signal-regulated kinase signalling (24). However, the levels of gluconolactone were not greatly increased in the non-survivors of the cardiac dysfunction group. According to relevant literature and our results, we speculate that the alteration of gluconolactone might be a protective adaptive response to overcome the cardiotoxic effects of sepsis.

Another important finding is that the level of galactitol was markedly increased in the non-survivors of the cardiac dysfunction group. ROC analysis demonstrated that it is an independent risk factor to predict 28-day mortality in cardiac dysfunction patients. Galactitol, a downstream metabolite of galactose metabolism, is generated by the hydrogenation of galactose *via* aldose reductase and cannot be further metabolized (25). A study also reported that galactitol emerged as the most obvious differential product in sepsis-related liver injury, suggesting that galactose metabolites are related to liver injury in the endotoxaemic state (26). However, the underlying mechanisms of galactitol and sepsis-induced organ dysfunction need further discussion. Under physiological conditions, aldose reductase has relatively low affinity for galactose. In the case of galactose accumulation, the production of galactitol increases, and multiple-aspect damage is induced. Galactitol leads to the exhaustion of nicotinamide adenine dinucleotide phosphate and the reduction of glutathione reductase activity and then acts as a metabolic toxin to the body and induces the accumulation of free radicals (25). Galactitol is incapable of diffusing across cellular membranes because of poor liposolubility, resulting in an increase in intracellular osmotic pressure (27). The free radicals together with the osmotic effect of galactitol eventually result in rupture of the cellular membrane and impairment of mitochondrial DNA and proteins (28). Further studies are needed to verify these potential mechanisms in sepsis.

Particular changes in the metabolic pathway occurred in this study. In the cardiac dysfunction group of septic patients, we found that the altered pathways were mainly involved in tryptophan metabolism, lysine degradation, and pentose phosphate pathway. In the non-survivors of the cardiac dysfunction group, we found that the changed pathways were mainly involved in galactose metabolism, primary bile acid biosynthesis and phenylalanine metabolism. Tryptophan metabolism, lysine degradation and phenylalanine metabolism belong to amino acid metabolism pathways. Both pentose phosphate pathway and galactose metabolism belong to the glucose metabolism pathways. Primary bile acid biosynthesis is associated with bile acid metabolism. Among these, tryptophan metabolism deserves special attention. Kynurenic acid, a candidate biomarker for diagnosing sepsis-induced cardiac dysfunction and predicting outcome, is an important metabolite of tryptophan metabolism. An increasing number of studies have revealed that tryptophan and its metabolites play key roles in inflammation-associated processes (29). In addition to influencing T-cell immunity and leading to immune tolerance, emerging studies have highlighted the pivotal role of tryptophan metabolism in modulating B-cell functions and humorall immunity (30). Aryl hydrocarbon receptor, a receptor that responds to tryptophan metabolites, affects the proliferation and switching of the immunoglobulin isotype in B cells (31). It has been reported that B cells and neutrophils regulate each other in bone marrow, and B cells modulate neutrophils’ tissue-damaging properties by influencing neutrophils in sepsis (32). Given the above, tryptophan metabolism has a major influence on clinical outcomes in critically ill septic patients.

Some limitations of our study should be considered. This study is a small, single-centre trial. Blood samples were analysed at only one time point. Further research with more participants will be performed to comprehensively evaluate the timing and dynamic changes in differential metabolites and metabolic pathways, providing a more precise view of changes during convalescence or deterioration. Our study also has some advantages. Currently, this is the first study to evaluate differential metabolites in septic patients with cardiac dysfunction. Patients were prospectively observed until 28 days after emergency admission. Mitochondrial function has a close relationship with cardiovascular disease. Future studies should include correlation analysis between the differential metabolites and mitochondrial bioenergetics and homeostasis to explore the underlying mechanism.

## Conclusion

We used metabolomics technique to demonstrate that the metabolites of patients with sepsis-induced cardiac dysfunction change substantially and are mainly associated with the metabolic pathways of amino acid metabolism, glucose metabolism and bile acid metabolism. We also clearly distinguished septic patients with and without cardiac dysfunction using metabolites. As a consequence, kynurenic acid and gluconolactone are candidate biomarkers for diagnosing sepsis-induced cardiac dysfunction; kynurenic acid and galactitol are candidate biomarkers for predicting 28-day mortality. Kynurenic acid is a common differential metabolite in the two analyses and could be used as a diagnostic and prognostic biomarker. The differential metabolites and pathways may also be useful as targets for the development of new therapies for septic patients with cardiac dysfunction.

## Data availability statement

The original contributions presented in the study are included in the article/**Supplementary Material**. Further inquiries can be directed to the corresponding authors.

## Ethics statement

The studies involving human participants were reviewed and approved by the Ethics Committee of Hunan Provincial People’s Hospital. The patients/participants provided their written informed consent to participate in this study.

## Author contributions

Conceptualization, YC, WX and XH. Methodology, YC and ZL. Formal analysis, YC and ZL. Investigation and data curation, YJ and WX. Writing—original draft preparation, YC, YP and WM. Writing—review and editing, CF and JH. Funding acquisition, YC, XH. WX and XH are the master investigators of the manuscript, take responsibility for guiding the whole work, from start to publication article. All authors contributed to the article and approved the submitted version.
