# Supplementary material for: Untargeted metabolomic profiling of sepsis-induced cardiac dysfunction
Source: Front Endocrinol (Lausanne). 2023 Feb 16;14:1060470. doi: 10.3389/fendo.2023.1060470 (PMC9978788; doi:10.3389/fendo.2023.1060470)
Supplement: Supplementary file 1 [file DataSheet_1.doc]

Supplementary Table 1 Binary logistic regression analysis of metabolites associated with

sepsis-induced cardiac dysfunction

| Variables | Odds ratio | 95% Confidence Interval | *P* value |
| --- | --- | --- | --- |
| Kynurenic acid | 1.602 | 1.039-2.471 | 0.033 |
| Gluconolactone | 1.348 | 1.014-1.793 | 0.040 |
| 3-Hydroxy-N6,N6,N6-  trimethyl-L-lysine | 0.849 | 0.726-0.993 | 0.040 |
| 25-Hydroxycholesterol | 0.918 | 0.863-0.977 | 0.007 |

Supplementary Table 2 Areas under the receiver operating characteristic(ROC) curves of metabolites associated with sepsis-induced cardiac dysfunction

| Variables | AUC | SE | 95% Confidence Interval | *P* value |
| --- | --- | --- | --- | --- |
| Kynurenic acid | 0.801 | 0.049 | 0.705-0.897 | ＜0.001 |
| Gluconolactone | 0.754 | 0.053 | 0.649-0.858 | ＜0.001 |
| 3-Hydroxy-N6,N6,N6-  trimethyl-L-lysine | 0.668 | 0.061 | 0.547-0.788 | 0.010 |
| 25-Hydroxycholesterol | 0.315 | 0.060 | 0.196-0.433 | 0.004 |

Supplementary Table 3 Differential metabolic pathways and enriched compounds between the normal cardiac function group and cardiac dysfunction group

|  | Total in  pathway | In set | Raw *P* | -log(*P*) | Enriched compounds（matching IDs） |
| --- | --- | --- | --- | --- | --- |
| Tryptophan metabolism | 83 | 3 | 0.019677652 | 1.706026714 | C01717  Kynurenic acid  C03722  Quinolinic acid  C05660  5-Methoxyindoleacetate |
| Pentose phosphate pathway | 35 | 3 | 0.024742548 | 1.606555574 | C00198  Gluconolactone  C00257  Gluconic acid  C00502  D-Xylonic acid |
| Lysine degradation | 50 | 2 | 0.047785401 | 1.320704764 | C00431  5-Aminopentanoic acid  C01259  3-Hydroxy-N6,N6,N6-trimethyl-L-lysine |

Total in pathway: total number of metabolites involved in the pathway

In set: the number of differential metabolites involved in the pathway

Raw *P*: *P* value for the metabolic pathway

-log(*P*): logarithm of the reciprocal of *P* value based on 10

Supplementary Table 4 Binary logistic regression analysis of metabolites associated with 28-day mortality

in the cardiac dysfunction group

| Variables | Odds ratio | 95% Confidence Interval | *P* value |
| --- | --- | --- | --- |
| Kynurenic acid | 1.162 | 1.001-1.349 | 0.048 |
| Galactitol | 1.416 | 1.954-2.101 | 0.046 |

Supplementary Table 5 Areas under the receiver operating characteristic(ROC) curves of metabolites associated with sepsis-induced cardiac dysfunction

| Variables | AUC | SE | 95% Confidence Interval | *P* value |
| --- | --- | --- | --- | --- |
| Kynurenic acid | 0.821 | 0.066 | 0.692-0.950 | 0.0017 |
| Galactitol | 0.773 | 0.084 | 0.608-0.937 | 0.0075 |

Supplementary Table 6 Differential metabolic pathways and enriched compounds between the survivors and non-survivors in cardiac dysfunction group

|  | Total in  pathway | In set | Raw *P* | -log(*P*) | Enriched compounds（matching IDs） |
| --- | --- | --- | --- | --- | --- |
| Galactose metabolism | 46 | 4 | 0.013672263 | 1.864159604 | C00089  Sucrose  C00137  myo-Inositol  C00794  Sorbitol  C01697  Galactitol |
| Primary bile acid biosynthesis | 47 | 3 | 0.016784672 | 1.77508715 | C01921  Glycocholic acid  C05122  Taurocholic acid  C05465  Taurochenodesoxycholic acid |
| Phenylalanine metabolism | 60 | 3 | 0.027734556 | 1.556978782 | C00805  Salicylic acid  C01586  Hippuric acid  C04148  Phenylacetylglutamine |

Total in pathway: total number of metabolites involved in the pathway

In set: the number of differential metabolites involved in the pathway

Raw *P*: *P* value for the metabolic pathway

-log(*P*): logarithm of the reciprocal of *P* value based on 10
